# Online Assessment of Human-Robot Interaction for Hybrid Control of Walking

**DOI:** 10.3390/s120100215

**Published:** 2011-12-27

**Authors:** Antonio J. del-Ama, Juan C. Moreno, Àngel Gil-Agudo, Ana de-los-Reyes, José L. Pons

**Affiliations:** 1 Biomechanics and Technical Aids Department, National Hospital for Spinal Cord Injury, Finca La Peraleda S/N, Toledo 45007, Spain; 2 Bioengineering Group, National Research Council (CSIC), Carretera Campo Real km 0.200, Arganda del Rey 28500, Spain

**Keywords:** human-robot interaction, hybrid control, gait, exoskeleton, functional electrical stimulation, muscle fatigue

## Abstract

Restoration of walking ability of Spinal Cord Injury subjects can be achieved by different approaches, as the use of robotic exoskeletons or electrical stimulation of the user’s muscles. The combined (hybrid) approach has the potential to provide a solution to the drawback of each approach. Specific challenges must be addressed with specific sensory systems and control strategies. In this paper we present a system and a procedure to estimate muscle fatigue from online physical interaction assessment to provide hybrid control of walking, regarding the performances of the muscles under stimulation.

## 1. Introduction

Spinal cord injuries result in impaired motor and sensory capacities. Gait impairments are frequent consequences of spinal cord injuries and can result in partial or total absence of voluntary control of the lower limbs. A common method used to partially recover standing and walking functions is repetitive rehabilitation training over several months. Task-specific repetitive training in conventional rehabilitation therapies has been demonstrated to be beneficial. Robotic exoskeletons have been widely proposed as instruments for gait therapy [1–4].

First generations of rehabilitation robotics for gait training have been designed to allow for repetitive training over longer sessions. Further improvements have been focused on increasing the active role of the patients. This has been mainly solved by allowing the users to deviate from predefined trajectories while providing visual and force feedback.

Hybrid control of walking consists of the combination of motorized exoskeletons and electrical stimulation of the patient’s muscles. With this approach, artificial control of biological muscle reduces the power demand of a motorized exoskeleton and more importantly, promotes several benefits in terms of increased muscle strength and cardiorespiratory fitness. Hybrid control of walking can be then defined as the technique aimed to compensate and/or rehabilitate gait by means of delivering and controlling power to the lower limb joints where total joint power is the result of induced muscle contraction and electromechanical actuation. Hybrid control of walking requires a method for online assessment of human-robot interaction which takes into account the effects of muscle fatigue. This assessment is required for continuous generation of biofeedback values for control according to the performance of biological and artificial actuators to the specific demands of the training task. Recently developed exoskeletons that allow for repetitive delivery of physiological gait patterns to patients have been equipped with sensors and computerized feedback to give precise information to the clinician and patients regarding the performance at each gait cycle. In [5] a method to calculate quantitative biofeedback values based on man-machine interaction forces is presented. Deviation with respect to reference profiles of interaction are proposed as means to calculate patient’s degree of activity. Force-based biofeedback method is proposed in [6,7]. In this method, values calculated are average values of forces measured within the exoskeleton while it drives the leg according to the gait cycle phase. Some researchers use ground reaction forces as a biofeedback source [4]. The center of pressure is determined during gait and used to adjust the assistance during the rehabilitation procedure. Regarding muscle fatigue, a recent study has attempted to extract muscle fatigue from superficial electrical myography (EMG), concluding that muscle fatigue could not be predicted by EMG [8]. Therefore other methods are need to estimate muscle performance under stimulation.

The aim of this paper is to present the design and preliminary study of online computerized assessment of the human-robot interaction for a hybrid gait trainer. The challenging design of force sensing in hybrid exoskeletons and the method for online computerized assessment of interaction are presented.

## 2. Methods

In a context of a more ambitious project, the target group of Spinal Cord Injured to whom the hybrid exoskeleton is directed are those whose lesion is referred as *Conus Medularis* [9], an incomplete Spinal Cord Injury. Functional aspects of this type of lesion that affect user’s walking function are the following:

1. Hip flexor muscle (psoas) function is preserved, so the subjects can flex the hip.
2. The capability to generate torque at knee joint varies among subjects.
3. There is an absence of volitional control over ankle joint.

### 2.1. Mechanical Design

The functional characteristics drives the exoskeleton design process. Only knee and ankle joint are actuated, given the preservation of hip flexors. The exoskeleton can be used by subjects up to 90 kg. The exoskeleton frame is based on a bi-lateral structure hinged at the knee and ankle joints, with a single external support bar, made of Aluminium 7075. It is customizable to the user due to telescopic rods built in the distal and proximal segments. Transmission of forces between the subject and the exoskeleton is secured by four girths and velcro straps, two at the thigh and two at the shank respectively.

The knee joint mechanism is made up of four-bar bio-inspired mechanism [10,11]. This mechanism allows the joint axis follow the translational motion of the physiological knee joint axis, improving kinematic compatibility between the orthosis and the subject with respect to a single-axis joint. This mechanism was modified to incorporate the actuator, comprising a DC flat motor (Maxon EC-90) and an Harmonic-Drive transmission (ratio 100:1). Motor and transmission were selected using non-pathological gait data at slow speed (0.5 m/s [12]), from which maximum torque and power to drive the knee were extracted.

**Figure 1.**
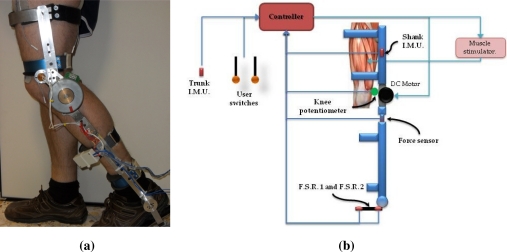
Hybrid exoskeleton prototype. **(a)** Real prototype; **(b)** Scheme of the sensory system.

The ankle joint actuation is directed to avoid the droop foot during swing phase of walking. This function is implemented by a torsional spring, which allows a lightweight and compact design. The stiffness of the spring has been calculated from anthropometric data particularized for a 90 kg user [12], in order to avoid drop foot during the swing phase, which resulted in 1.4 Nm/°. Such stiffness allows plantar and dorsal flexion during stance phases of gait with a low interference with the movement. The exoskeleton prototype is shown in Figure 1(a).

### 2.2. Sensory System Design

Most of the sensory system is aimed to monitor the events and states of walking. As stated in Section 2.4, online interaction analysis makes necessary to complete the sensory system with a force sensor, which is shown in Section 2.3. An scheme of the hybrid exoskeleton’s sensory system is shown in Figure 1(b).

Normal gait can be described as a series of states and transitions among them. Main states are *stance*, when the foot is contacting the floor, and *swing*, when the foot does not contact with the floor. These two main states impose different kinematic joint trajectory and stiffness to the exoskeleton joint actuators so the controller must be fed with the information regarding foot contact with the floor. Therefore we have implemented two *Force Sensing Resistors* (FSR) (Interlink Electronics Inc., 38.1 mm × 38.1 mm), placed at the heel and tip of the exoskeleton’s foot insoles, which provides an ON/OFF signal to the controller to discriminate between swing and stance.

As mentioned, each gait state imposes a kinematic reference to the exoskeleton’s joint actuator, so it is necessary to monitor the actual joint angle. In our design, only knee joint is actively controlled, while ankle joint is passively controlled by a torsional spring. Monitoring of knee angle is done trough a rotational potentiometer (Vishay Espectrol). In order to assess gait biomechanics, we measure also shank and trunk orientation as well as hip joint angle. Orientation of shank segment is measured by an inertial measurement unit (TechIMU, TechnAid S.L.), which gives the 3D orientation in real time. This inertial measurement unit (IMU) uses information from a 3D accelerometer, a 3D gyroscope and a 3D magnetometer to calculate the orientation of the IMU in a world-referenced coordinate system. Another IMU is used to measure trunk orientation and, combined with the shank IMU, we calculate the 3D hip angle. Also, the trunk IMU is used to monitor trunk orientation, in order to provide information about user’s equilibrium and intention: if the trunk is tilted forward, the subject aims to keep walking, while if the trunk is extended or tilted backward, the subject aims to stop walking.

This set of sensors, in combination with the knee actuator, the ankle passive actuator and an adequate control strategy, allows to achieve an automatic walking. However, it has been described that the users of this technology does not feel comfortable with this kind of automatic behavior [13]. Therefore, volitional control of the hybrid system is accomplished by two hand-switches, implemented by a small force-sensing resistor. This implementation allows placing a hand switch on the user’s grip of crutches or walker without interfering with it. Orders implemented are: sit to stand, right leg step, left leg step, stand, and stand to sit. Those orders are combined with the information form the inertial sensor placed at the trunk to avoid dangerous situations (*i.e.*, order of “step” when the trunk is tilted backwards)

### 2.3. Force Sensor Design

Extracting information regarding physical interaction can be achieved via a force sensor placed on the exoskeleton structure. The interaction force is the force developed at the leg-exoskeleton interface during the movement, that is, the girths’ inner surface over the leg skin. This force, depending on the distance at the girths are placed for a specific subject, can be transformed to a torque at the knee joint level.

There is a broad offer of commercially available force sensors. However, it is difficult to find an adequate force sensor, with adequate geometry and range of measure, that can be placed at the exoskeleton structure. Moreover, integration of a force sensor will result on introduction of discontinuities on the structure. Therefore, we instrumented the structure to turn the structure into the force sensor itself.

In order to instrument the structure, we have introduced a full gauge bridge over its distal part, below the knee joint and over the first girth of the distal segment of the exoskeleton. Measurement of forces through strain gauge is based on measuring the deformation of the structure caused by the own force [14]. Therefore, the exoskeleton structure must deform adequately in order to measure the interaction torque. Of course, this deformation should be enough to provide adequate resolution, but must not be excessive to not overcome the strain limit of the aluminium.

We performed an analysis of the exoskeleton structure in order to design most adequate geometry to achieve a desirable deformation. This analysis had a two-fold objective: first, the geometry would should allow to place a full gauge bridge. Second, an adequate range of measure must be achieved, related with the forces involved during walking. An estimation of knee joint moment during non-pathological gait at low speed [12] was taken to perform the analysis. Other conditions assumed were the following:

1. Muscle electrical stimulation will develop 25% of maximum knee joint torque during walking.
2. Position of shank straps is the one that maximize the deformation of the structure.
3. Transverse stiffness must be maximized.

**Figure 2.**
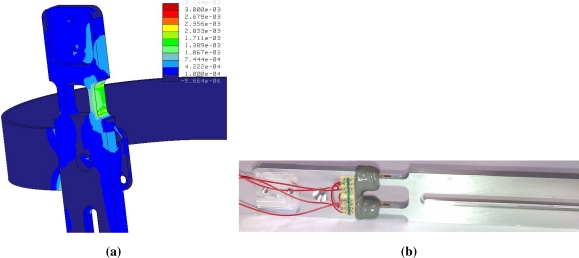
Force sensor. **(a)** FEM analysis result: maximum deformation during extension; **(b)** Actual sensor: full bridge implementation.

Once the geometry was designed, a verification by finite-element analysis was conducted. A maximum strain of 0.2% was obtained (Figure 2(a)). The actual sensor is shown in Figure 2(b).

### 2.4. Human-Robot Physical Interaction Assessment

**Figure 3.**
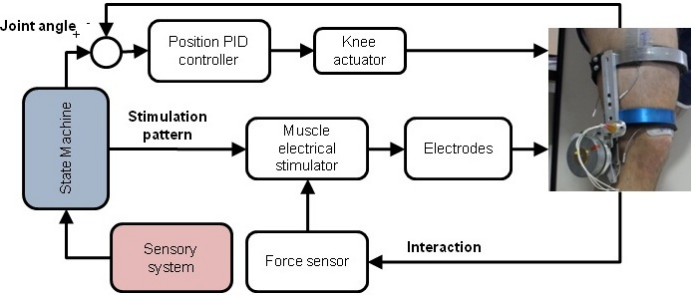
Hybrid control architecture.

Our hybrid approach for control of walking is based on monitoring the physical interaction between the user and the exoskeleton. Although the design and implementation of the control architecture is out of the scope of this article, it is necessary a brief description. It is shown on Figure 3. The control architecture has two control loops: one performs closed loop control of knee trajectory (top loop at Figure 3), and the other control the muscle stimulator. Coordination between the two controllers (therefore the hybrid control) is performed by the state machine, which governs both controllers. This state machine (blue box) imposes kinematic pattern to the PID controller of the knee, and the stimulation pattern (defined by channel to activate and its characteristics of intensity, frequency and pulse width) to the stimulator, regarding the information given by the sensory system.

Closed loop control of muscle stimulation is difficult to perform due to the absence of appropriate signals from the muscle under stimulation that allow to perform direct closed loop control of the stimulation parameters. Instead indirect measures have been taken to close the stimulation control loop, such as joint angle due to the stimulation [15,16], the interaction torque developed due to muscle stimulation [17,18] or a combination of joint angle and interaction torque [19,20], to adjust stimulation intensity. However, none of these work has directly managed muscle fatigue due to stimulation, therefore system’s efficacy would decrease. Our approach aims to close the stimulation loop by monitoring muscle performance through user-exoskeleton physical interaction. This information will be used to modulate stimulation intensity regarding muscle performance.

### 2.5. Experimental Setup

We performed a preliminary experiment with a healthy subject in order to corroborate that our design is able to assess human-robot interaction for fatigue estimation. Muscle performance is assessed from physical user-exoskeleton interaction under the hypothesis that user’s joint torque developed by muscle electrical stimulation will decrease due to fatigue, as the muscle will generate less force with time while the stimulation pattern (intensity, pulse width and frequency) is kept unchanged. This reduction in muscle force must be counterbalanced by the exoskeleton actuator, applying more force to the leg, which will result in an increase of the interaction torque. Therefore, interaction torque can be used to estimate muscle fatigue due to electrical stimulation.

In order to have a more robust muscle fatigue estimate than instantaneous interaction torque, we have developed a metric that would represent the mean interaction torque produced during a time interval. To provide an adequate basis of comparison, we have focused only on the swing phase of gait because, considering also that the stance phase would introduce uncertainties into force readings as the user can support its own weight with the other leg (or crutches if it is a SCI user), which may vary among steps. Therefore, as the muscle fatigue index is applied to swing phase, the experiments performed were focused on such phase.

This mean interaction torque is calculated by equations (1) (mean interaction torque during leg flexion) and (2) (mean interaction torque during leg extension). In a first approach, we have analyzed the mean interaction torque during flexion and extension separately given that the muscles involved in each movement are different.

(1) $$\bar{T_{\mathrm{flex}}}=\frac{\int_{\mathrm{flex}}T_{\mathrm{int}}\cdot \mathrm{dt}}{t_{\mathrm{flex}}}$$

(2) $$\bar{T_{\mathrm{ext}}}=\frac{\int_{\mathrm{ext}}T_{\mathrm{int}}\cdot \mathrm{dt}}{t_{\mathrm{ext}}}$$

In those expressions, $\bar{T_{\mathrm{flex}}}$ and $\bar{T_{\mathrm{ext}}}$ are the mean interaction force, calculated by integrating the interaction force measured by the sensor (*T_int_*), during both flexion and extension intervals, averaged by the interval time (*t_flex_* and *t_ext_*, respectively). This subject did not practice sports at least for 24 h prior to the experiment. The experiments lasted 10 min, with the leg free to swing. User was requested to not to move the leg and try to relax during the experiment, in order to avoid any voluntary movement. The kinematic pattern fed to the controller was a series of flexion-extension trajectories from a non-pathologic gait kinematics database, that is, only the swing phase of gait. This kinematic pattern was slightly accelerated to shorten the experiment duration.

**Figure 4.**
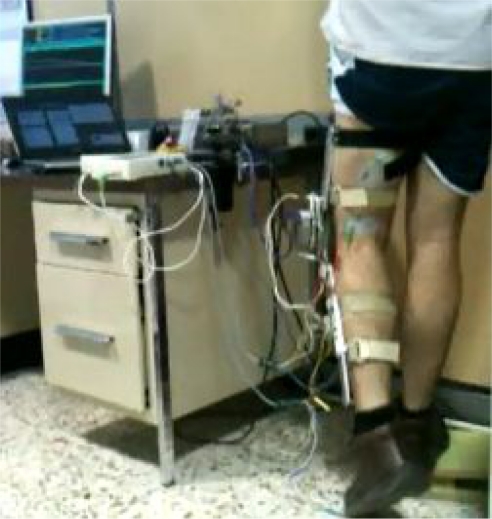
Experiment setup.

While the exoskeleton controls the joint trajectory, a synchronized stimulator (Unafet8) delivered stimulation pulses to *vastus lateralis* muscle during extension and *biceps femoris* muscle during flexion. Stimulation pulses was monophasic compensated, 300 ms pulse width and 40 Hz frequency. Stimulation intensity was set regarding user comfort perception, but high enough to achieve a functional knee joint movement. The intensity achieved was 46 mA for flexors muscles and 28 mA for extensors muscles. The delivered stimulation pulses were synchronized with the kinematic pattern. Figure 4 shows the experiment setup.

## 3. Results

**Figure 5.**
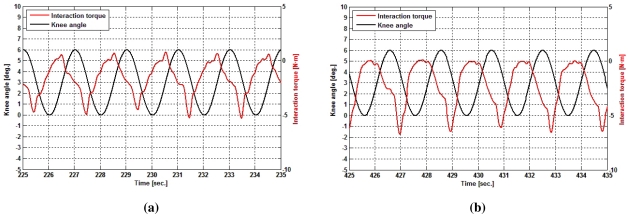
Knee joint kinematics and interaction torque during an experiment. **(a)** Middle cycles of the experiment; **(b)** Last cycles of the experiment.

Figure 5 displays two different time intervals of the experiment. In these figures are shown both the evolution of the knee joint angle (black curve) and the interaction torque at knee due to interaction torque (red curve). Figure 5(a) shows the evolution at the middle of the trial, while Figure 5(b) shows the last part of it. Comparing both figures, a change can be seen in the interaction strength, namely a decrease in maximum values and also the envelope of the curve itself, while joint trajectory remains constant. This increase in the interaction with time can be attributed to muscle fatigue.

**Figure 6.**
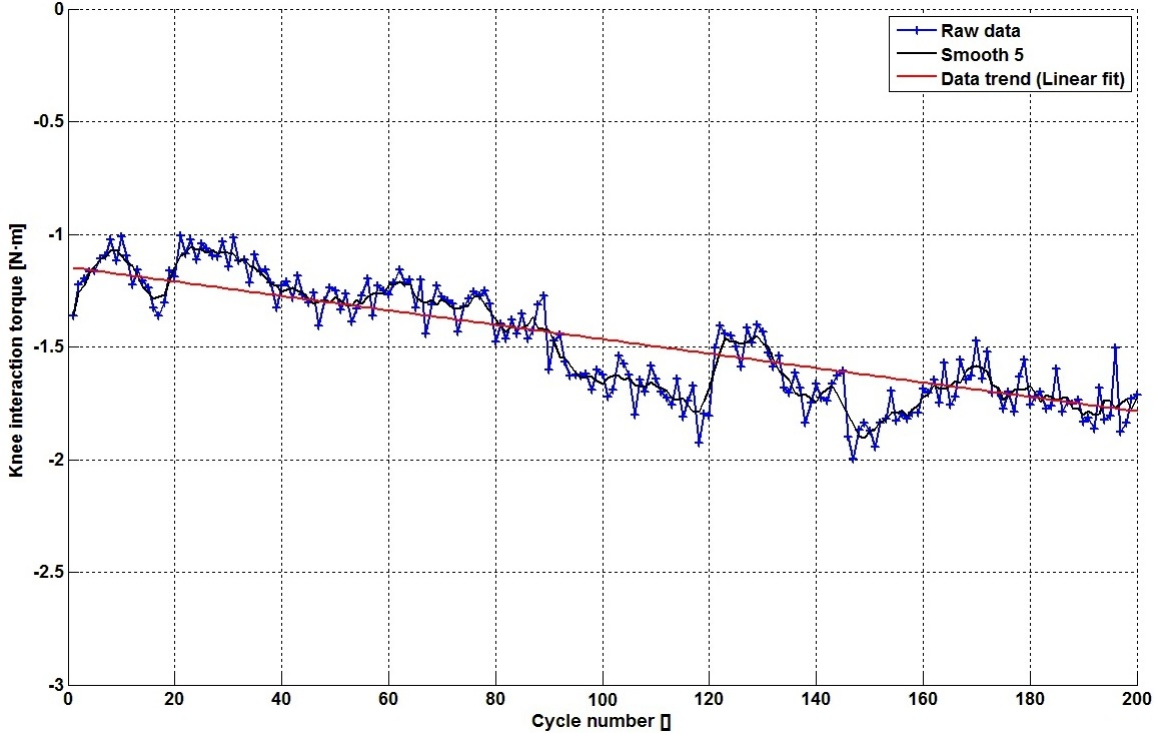
Mean interaction torque.

The change on the interaction torque can be observed more clearly through the application of the metrics defined in the equations (1) and (2). By applying these metrics during leg extension, we obtain the blue curve shown in Figure 6. This curve shows the average interaction torque at each swing cycle. Data displayed on this curve have certain variability, so a moving average of 5 samples improves data representation (Figure 6, black curve), in order to detect data trend (Figure 6, red curve). It can be seen an increasing trend of torque interaction in these data. When compared with Figure 5, values shown in Figure 6 provide more robust information about muscle performance due to fatigue.

**Figure 7.**
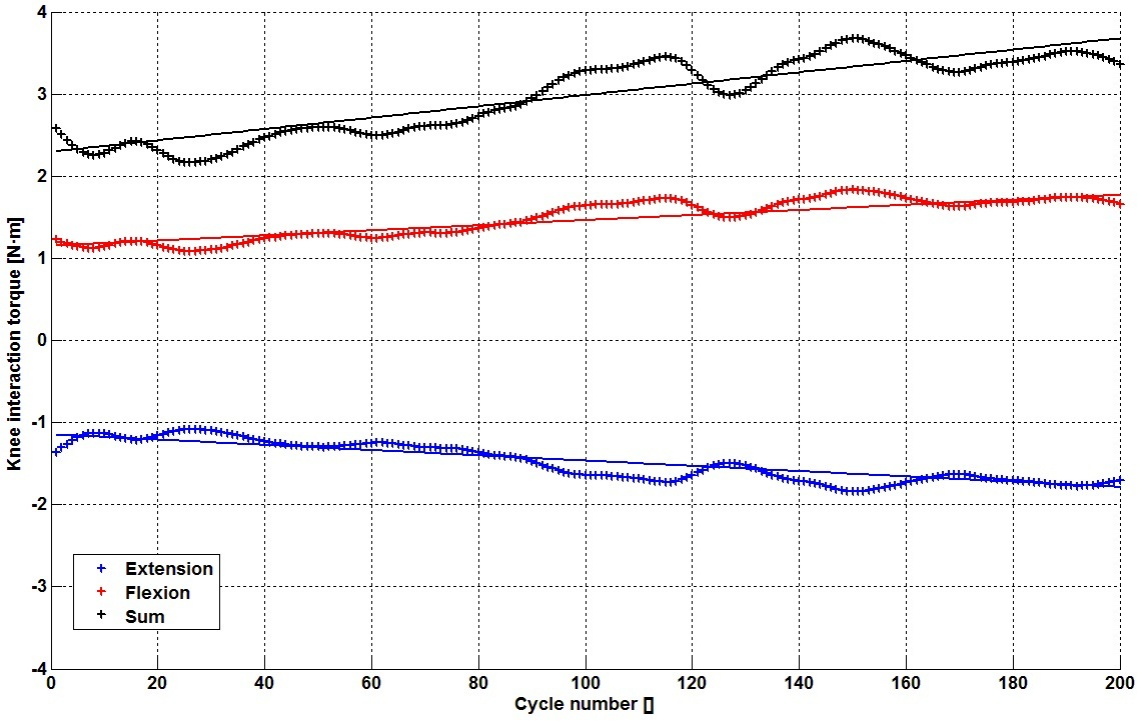
Muscle fatigue estimation metrics.

Figure 7 shows the application of this metric to both the flexion (red curve) and extension phase (blue curve). During flexion movement, the magnitude of the metric is highly similar to the extension phase, as both increase its magnitude. Taking the absolute value of each curve, both can be combined into a single metric that represents the interaction torque during the whole swing phase (Figure 7, black curve). Linear fit coefficients of fatigue trend are in Table 1.

## 4. Conclusions

In this article, we have presented a system and developed a procedure to assess human-robot physical interaction for Hybrid Control of Walking in spinal cord injured subjects. The sensory system allow online monitoring of user-robot physical interaction to assess muscle fatigue during swing phase of gait. This approach will be used during walking with the fatigue estimator active during the swing phase of gait, being inactive during stance phase and waiting to collect again data when next swing phase occurs. This estimation of muscle fatigue allows modulating muscle electrical stimulation, regarding muscle performance, which in turn would result in longer periods of muscle stimulation. Therefore, the user would benefit from longer periods of training and walking. Also, information from physical interaction can be used to implement a control strategy in which the exoskeleton controller can collaborate with a muscle stimulation controller.

We have shown that a decrease in muscle performance due to fatigue is immediately reflected in a decrease of interaction torque (or torque at joint level). In order to have an estimate of muscle fatigue, we have developed a metric which compares the mean interaction torque at each swing cycle. While considering both trajectories during swing phase (flexion and extension), we have obtained a similar behavior from both directions. Therefore, we can combine these values to give a more robust estimate. The work presented in this article substantiate our initial hypothesis that muscle fatigue due to muscle electrical stimulation can be estimated from interaction torque. However, from this point on, some points must be stated and solved in future work.

First, this preliminary experiment has been conducted with a healthy subject. Therefore these results must be validated with injured subjects, although given the weak muscular condition of such subjects, we expect that muscle fatigue would be even more clear than in a healthy subject. Second, the application to hybrid gait control must be validated. Hybrid control of gait will be performed using pre-defined muscle stimulation patterns available in the literature, while online assessing of muscle fatigue shown in this article will modulate stimulation intensity.

## Figures and Tables

**Table 1.** Linear fit coefficients.

Flexion	0.0031 Nm/cycle
Extension (abs.)	0.0032 Nm/cycle
**Total swing phase**	**0.0063 Nm/cycle**
